# The effect of biologging systems on reproduction, growth and survival of adult sea turtles

**DOI:** 10.1186/s40462-018-0145-1

**Published:** 2019-01-29

**Authors:** Lucy C. M. Omeyer, Wayne J. Fuller, Brendan J. Godley, Robin T. E. Snape, Annette C. Broderick

**Affiliations:** 1 Marine Turtle Research Group, Centre for Ecology and Conservation, College of Life and Environmental Sciences, University of Exeter, Penryn, Cornwall TR10 9FE United Kingdom; 2 Faculty of Veterinary Medicine, Near East University, Nicosia, Mersin 10, North Cyprus Turkey; 3 Society for the Protection of Turtles, PK.65 Kyrenia, Mersin 10, North Cyprus Turkey

**Keywords:** Animal welfare, Green turtles, Loggerhead turtles, Satellite tracking, Tagging effect, Tagging reflex, Telemetry systems

## Abstract

**Background:**

Telemetry and biologging systems, ‘tracking’ hereafter, have been instrumental in meeting the challenges associated with studying the ecology and behaviour of cryptic, wide-ranging marine mega-vertebrates. Over recent decades, globally, sea turtle tracking has increased exponentially, across species and life-stages, despite a paucity of studies investigating the effects of such devices on study animals. Indeed, such studies are key to informing whether data collected are unbiased and, whether derived estimates can be considered typical of the population at large.

**Methods:**

Here, using a 26-year individual-based monitoring dataset on sympatric green (*Chelonia mydas*) and loggerhead (*Caretta caretta*) turtles, we provide the first analysis of the effects of device attachment on reproduction, growth and survival of nesting females.

**Results:**

We found no significant difference in growth and reproductive correlates between tracked and non-tracked females in the years following device attachment. Similarly, when comparing pre- and post-tracking data, we found no significant difference in the reproductive correlates of tracked females for either species or significant carry-over effects of device attachment on reproductive correlates in green turtles. The latter was not investigated for loggerhead turtles due to small sample size. Finally, we found no significant effects of device attachment on return rates or survival of tracked females for either species.

**Conclusion:**

While there were no significant detrimental effects of device attachment on adult sea turtles in this region, our study highlights the need for other similar studies elsewhere and the value of long-term individual-based monitoring.

**Electronic supplementary material:**

The online version of this article (10.1186/s40462-018-0145-1) contains supplementary material, which is available to authorized users.

## Background

Telemetry and biologging systems, ‘tracking’ hereafter, have been instrumental in meeting the challenges associated with studying the ecology and behaviour of cryptic, wide-ranging marine mega-vertebrates [1]. Such systems have evolved greatly, particularly over the last two decades, becoming smaller, with increased storage capacity. Thus, they have provided scientists with a powerful tool, with which to obtain key information not previously available [2]. Technological advancements have permitted the tracking of smaller animals [3–5], across multiple life stages [6] and around the world [7, 8]. Although tracking has greatly furthered our understanding of the natural world, it is key to determine whether the data collected are unbiased and, whether derived estimates can be considered typical of the population at large.

Although benign in some instances [9–12], device attachment does not always come free of cost to study animals. For example, it can lead to increased energy expenditure [13–15], influence reproductive success [16–18] as well as alter natural behaviours [19–22]. Device improvements have led to the tracking of animals over extensive periods of time [23–25], which may have physiological implications [26], with potential carry-over effects [27, 28]. In addition, a recent meta-analysis on birds highlights that these effects may be cumulative, such that, for example, effects on annual survival could also impact reproduction [29]. Therefore, assessing the effects of device attachment on the overall fitness of study animals, in both the short- and long-term, focussing on multiple traits [29], is crucial to mitigate against potential deleterious effects in the future.

Over the years, tracking has increased exponentially, worldwide, across species and life-stages in sea turtles [30, 31], contributing widely to priority research questions [32]. It has allowed researchers to explore migration patterns [33], diving behaviours [34–36] and foraging strategies of sea turtles [37, 38], as well as providing improved estimates of sea turtle abundance [39, 40]. Despite this increase in use, the number of studies that consider ethical or welfare issues associated with device attachment is low 20%; [31], and the number of studies that investigate welfare issues as their main theme is even lower 2%; [31]. Device attachment has been modelled as increasing drag and energy expenditure [15, 41], potentially influencing reproductive correlates and survival of study animals. Empirically, differences in swimming efficiency and diving capacities, as well as differences in data quality, have been reported in leatherback turtles (*Dermochelys coriacea*) based on attachment methods alone [20, 21]. Such studies highlight the need for an evaluation of the effects of device attachment on life-history traits and survival of study animals, as well as the need to compare data between tracked and non-tracked individuals.

Here, we provide the first long-term analysis of the effects of device attachment on reproduction, growth and annual survival of green (*Chelonia mydas*) and loggerhead (*Caretta caretta*) turtles nesting sympatrically, using a 26-year individual-based monitoring dataset, with devices first attached in 1997. We compare differences in reproductive correlates, growth and annual survival, both between tracked and non-tracked females, and pre- and post-tracking of individual females.

## Methods

Further details for each corresponding section can be found in the Additional file 1 for this article.

### Study site and data collection

Data were collected at Alagadi beach, Northern Cyprus (35°33 N, 33°47 E) between 1992 and 2017, where intensive night-time monitoring and tagging programmes have been carried out (see [42] for detailed methods). Female identification was based on flipper tags and passive integrated transponder tags (PIT tags; [43]). Curved carapace length (CCL) notch to notch was used as a measure of female size. Growth was calculated from CCL measurements (see [44] for further details). Due to the intensive nature of the monitoring carried out at Alagadi, very few nests per year cannot be attributed to individual females [45]. However, when a missed nest was apparent (i.e. intervals of > 18 days observed between two clutches), clutch frequency was adjusted and referred to as ECF (estimated clutch frequency) hereafter (see [42, 45] for further details). Mean clutch size and ECF were calculated for each individual, each nesting season. Remigration interval (RI) was calculated as the number of years elapsed between two nesting seasons. Date of first nest was determined as the day of the year (d.o.y) the female was first observed laying. ECF and date of first nest were not calculated in 1992 due to incomplete survey effort.

### Device attachment

A variety of devices (Table 1) were attached to nesting females between 1997 and 2017, following the protocol outlined by Godley et al. [46]. Satellite transmitters (PTTs: platform terminal transmitters) were attached for studies of migration, whereas all other devices were designed to be recovered within a breeding season to investigate inter-nesting behaviours. Given that all devices were attached in a similar manner and were of similar magnitude, we consider animals with any devices attached as ‘tracked’, irrespective of device type. In some instances, multiple devices of the same type were attached within a breeding season using the same attachment base. For the analysis, however, we focussed only on the last attached device. Note that attaching multiple devices of the same type to the same base attachment platform is no different to attaching one device early in the nesting season, which is not retrieved between clutches. Although some females were fitted simultaneously with two devices, not all females returned to foraging grounds with both devices attached (Table 1). Except for PTTs, whenever possible, devices were retrieved, leaving behind the attachment base, except in 1997, when the base was also removed. Individuals for which the base was removed were included in this analysis of return rates but were excluded for the remainder of the analysis. Females that returned to foraging grounds without any devices attached were included in the analysis because, although the attachment base was shaped to reduce drag, it could not be excluded that it did not affect individuals. We distinguish between ‘tracked’ females with a device attached (hereafter referred to as ‘device attached’) and females for which only the attachment base remained (hereafter referred to as ‘attachment base only’).

**Table 1:** Devices attached to nesting females

Device type	Device weight (g; range)	Green turtles (*n* = 51)	Loggerhead turtles (*n* = 50)	References
PTT	275–750	26	25	[24, 46, 70, 89–91]
i-gotU® data loggers	37	24	33	[92]
GLS	48	20	13	[93]
TDR	16–200	16	5	[72, 73, 94–96]
PTT & GLS	162–275	4 ^a^	2 ^b^	[93]
GLS & Camera	700	2	0	[97]
Total	na	92	78	na

This table includes females that had devices attached in multiple years as well as within the same breeding season. *PTT* platform terminal transmitter, *GLS* global location sensing, *TDR* time depth recorder, *na* not applicable

^a^Female was fitted with one PTT and 3 GLS during the nesting season and returned to foraging grounds with both devices attached

^b^Female returned to foraging grounds with both devices attached

### Statistical analysis – Return rates

Fisher’s exact tests were used to calculate differences in return rates among groups, looking at differences between ‘tracked’ and ‘non-tracked’ as well as within ‘tracked’ groups.

Odds ratios were used as a measure of effect size. Females that were not resighted were assumed to be dead, although it is possible that individuals migrated to other nesting beaches which are not monitored during the night.

### Statistical analysis – Among-female differences

To investigate differences between ‘tracked’ and ‘non-tracked’ females, ‘initial year’, i.e. year of device attachment for ‘tracked’ females, was determined as the first year of capture for ‘non-tracked’ neophyte (first time nesters) females and was randomly generated for other ‘non-tracked’ females for which three or more captures were available. This means that the analysis only included females for which two or more captures were available.

One-way analysis of variance and linear models were used to compare differences in body size and reproductive correlates among groups. The analysis was conducted in R version 3.2.3. Models were fitted by stepwise model simplification and significance of removed terms was assessed with a threshold of *P* = 0.05 [47]. We checked for over-dispersion, normality, homoscedasticity and homogeneity of variance. Female size and ‘year’ were included as fixed effects to control for larger females laying larger clutches [42] and to investigate whether differences were due to annual effects. Partial omega squared ω_p_^2^ was used as a measure of effect size. Tukey post-hoc tests were used to look at pairwise comparisons, using the package multcomp [48]. Furthermore, we looked at the interactions between growth covariates and device attachment to investigate whether device attachment influenced growth of ‘tracked’ females (see [44] for further details).

### Statistical analysis – Within-female differences

Generalised linear mixed models (GLMM) and generalised least squares (GLS) were used to investigate within-female differences in reproduction between pre- and post-tracking years. To detect small non-significant effects of device attachment, a systematic analysis was used to look at seasonal (mean clutch size*ECF) and annual reproductive output (seasonal reproductive output/RI). Models were implemented using nlme and mgcv packages [49, 50] and included female identity as a random effect and ‘year’ as a fixed effect. CCL was also included in models of mean clutch size and seasonal/annual reproductive output. Models were fitted as explained in the previous section.

### Statistical analysis – Carry-over effects

To investigate whether device attachment had any carry-over effects, GLMM and GLS were used on a subset dataset that included only females for which two pre- (including year of device attachment) and two post-tracking seasons were available. This restricted the analysis to nine green turtle females, with ‘attachment base only’ and ‘device attached’ groups pooled. Sample size was too small for loggerhead turtles (*n* = 4). Models were fitted as explained in the previous section.

### Statistical analysis – Survivorship estimates

Encounter histories were created based on successful nesting attempts. Survival probability was estimated using the multi-state model in the programme MARK [51], assuming a breeding state (B; observable state) and a non-breeding state (NB; unobservable state). The parameters estimated were survival probability (S), encounter probability (p) and transition probabilities between states (ψ_B → NB_ and ψ_NB → B_). Goodness of fit was assessed using the programme U-CARE [52]. In particular, test component 3G.SR was used to evaluate the effect of presumed transient individuals on survival probabilities and, test component M.ITEC was used to test for trap-dependence. Transient individuals are individuals that are caught, marked and released but never recaptured. Such individuals can be considered in transit and therefore have a zero probability of recapture although they are alive. Model selection was based on the lowest qAIC_c_ value (corrected quasi-likelihood Akaike information criterion). Parameters were estimated using the Markov chain Monte Carlo method in MARK. Parameters estimates were based on posterior distributions and 95% highest posterior density credibility intervals were reported.

## Results

### Return rates

A total of 170 devices (Table 1) were attached to 51 green and 50 loggerhead turtle females between 1997 and 2017. Of these females, 13 green and 9 loggerhead turtle females had devices attached in two different years and 3 green turtle females had devices attached in three different years. However, the remainder of the analysis focused on females that had devices attached in a single year.

Almost all green turtles (93%) and just under three quarters of loggerhead turtles (70%) that had devices attached in a single year were resighted within a maximum of 15 years (Table 2). Of the females that had devices attached in a single year, 17% of green and 48% of loggerhead turtles were neophyte females. For both species, there was no significant difference in return rates between groups (Table 2 and Additional file 1: Table S1). Based on the odds ratios, ‘tracked’ neophyte females were no more or less likely to be resighted than ‘non-tracked’ neophyte females or ‘tracked’ remigrant females (Additional file 1: Table S1). Similarly, ‘attachment base only’ females were no more or less likely to be resighted than ‘device attached’ females (Additional file 1: Table S1).

**Table 2:** Returns rates for tracked and non-tracked females

Species	Non-tracked			Tracked		
Groups						
	≤ 5 yr	≤ 10 yr	≤ 15 yr	≤ 5 yr	≤ 10 yr	≤ 15 yr
Green turtles						
All females	na	na	na	76% (*n* = 45)	86% (*n* = 43)	93% (*n* = 40)
Neophytes	39% (*n* = 236)	51% (*n* = 197)	53% (*n* = 189)	63% (*n =* 8)	75% (*n =* 8)	86% (*n* = 7)
Remigrants	na	na	na	78% (*n* = 37)	89% (*n* = 35)	94% (*n* = 33)
‘Attachment base only’	na	na	na	84% (*n* = 19)	89% (*n =* 19)	89% (*n =* 19)
‘Device attached’	na	na	na	69% (*n* = 26)	83% (*n* = 24)	95% (*n* = 21)
Loggerhead turtles						
All females	na	na	na	47% (*n* = 30)	50% (*n* = 28)	70% (*n* = 20)
Neophytes	21% (*n* = 387)	29% (*n* = 325)	34% (*n* = 274)	33% (*n* = 9)	38% (*n =* 8)	60% (*n =* 5)
Remigrants	na	na	na	52% (*n =* 21)	55% (*n =* 20)	73% (*n* = 15)
‘Attachment base only’	na	na	na	100% (*n* = 6)	100% (*n =* 6)	100% (*n =* 6)
‘Device attached’	na	na	na	33% (*n =* 24)	36% (*n* = 22)	57% (*n* = 14)

Percentage of females resighted after 5, 10, 15 or less years after device attachment or for non-tracked females for both green and loggerhead turtles. All of the resighted females were resighted within a maximum of 15 years. *na* not applicable

### Among-female differences

The basic parameters for each group and species are summarised in Additional file 1: Table S2.

#### Green turtles

**Fig. 1:**
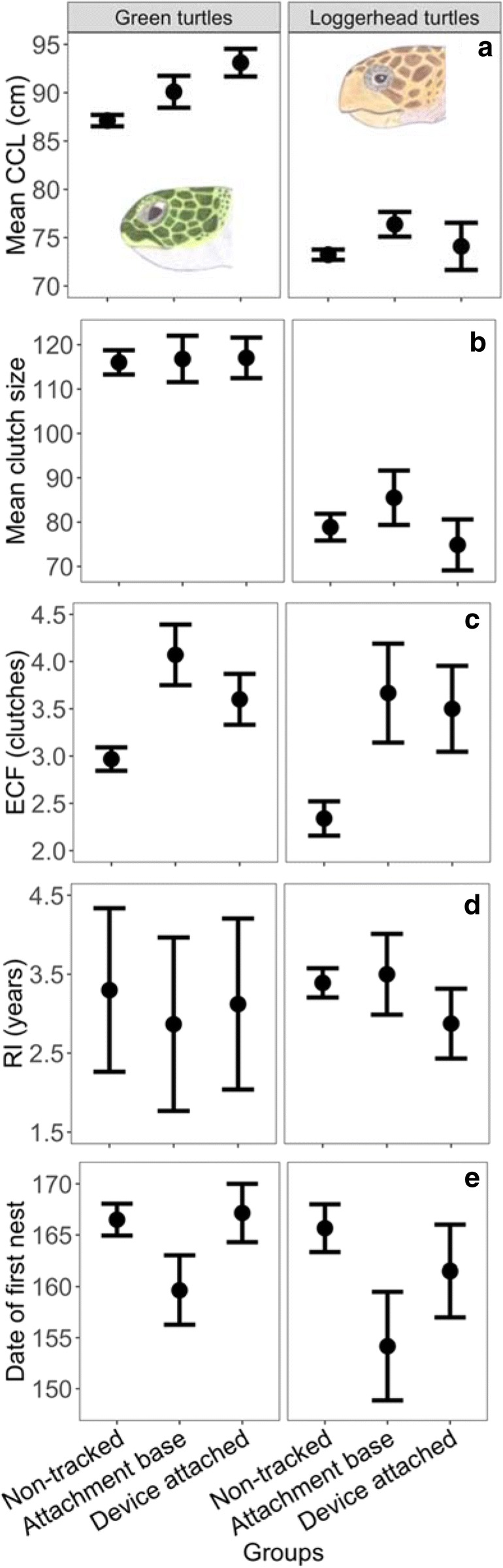
Effects on reproduction among females. Differences in female size (**a**) in the year of device attachment and reproductive correlated in the year following device attachment (**b**-**e**) for groups of females. For ‘non-tracked’ females, year(s) of and following device attachment represent randomly generated following recaptures. Observed differences in mean clutch size (eggs) for green turtles and in date of first nest (d.o.y) for both species were due to annual effects rather than device attachment (see main text). Mean ± SE. CCL: curved carapace length; ECF: estimated clutch frequency; RI: remigration interval; d.o.y: day of the year

For green turtles, there was a large significant difference in body size in the year of device attachment among groups (F_2,125_ = 9.30, *P* < 0.001, ω_p_^2^ = 0.115, Fig. 1a), with ‘device attached’ females being on average 6.0 cm (95% CI: 2.6–9.4, P_adj_ < 0.001, *n* = 20) larger than ‘non-tracked’ females (*n* = 94). There was, however, no significant difference in body size between ‘device attached’ and ‘attachment base only’ females (P_adj_ = 0.416) and between ‘non-tracked’ and ‘attachment base only’ females (P_adj_ = 0.176, *n* = 14).

In addition, there was no significant difference in mean clutch size (F_2,125_ = 2.92, *P* = 0.058, ω_p_^2^ = 0.029, Fig. 1b), RI (F_2,125_ = 0.65, *P* = 0.586, ω_p_^2^ < 0.001, Fig. 1d) and date of first nest (F_2,125_ = 3.00, *P* = 0.053, ω_p_^2^ = 0.030, Fig. 1e) among groups in the years following device attachment. Observed difference in mean clutch size (F_1,126_ = 5.68, *P* = 0.019, ω_p_^2^ = 0.037, Fig. 1b) and date of first nest (F_1,126_ = 12.26, P < 0.001, ω_p_^2^ = 0.065, Fig. 1e) were due to annual effects rather than device attachment.

**Fig. 2:**
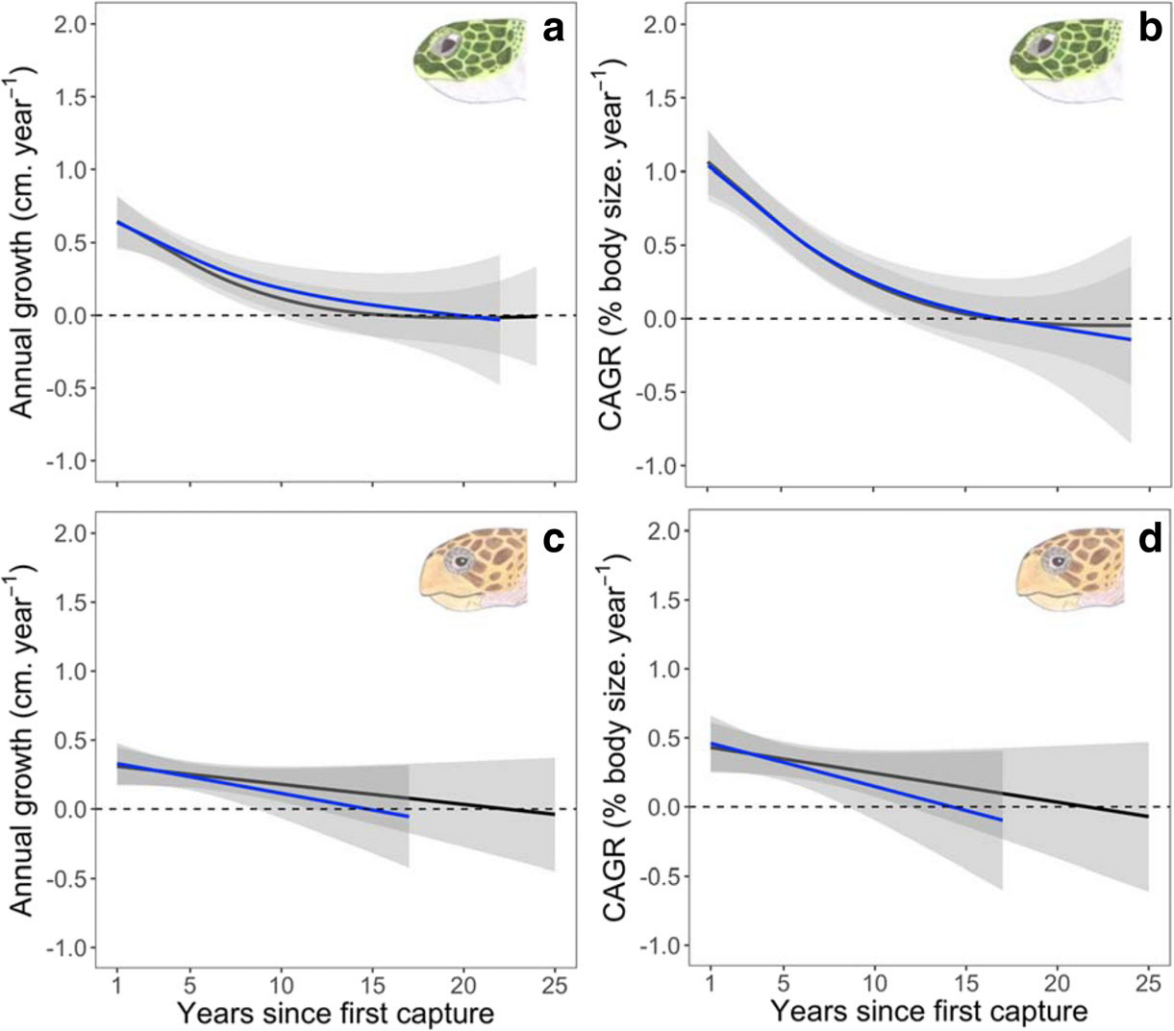
Effects on growth. Summary of (**a**, **b**) generalised additive mixed model and (**c**, **d**) generalised linear mixed model analyses of annual growth and compound annual growth rates (CAGR) for (**a**, **b**) green and (**c**, **d**) loggerhead turtles for the complete dataset (1992–2017) mentioned in Omeyer et al. [44]. The response variables are shown on the y axis, shifted by the intercept for ease of visualisation. Grey shaded area represents 95% confidence intervals. Dashed lines represent the absence of growth. The black lines represent the model outputs for growth records of ‘non-tracked’ and ‘tracked’ females pooled and represent the model outputs presented in Omeyer et al. [44]. The blue lines represent the models outputs for ‘non-tracked’ females and for ‘tracked’ females up until year of device attachment

However, there was a significant difference in ECF among groups in the years following device attachment (F_2,126_ = 6.528, *P* = 0.002, ω_p_^2^ = 0.085, Fig. 1c), which could not be explained by annual effects (F_1,125_ = 1.09, *P* = 0.297, ω_p_^2^ = 0.006). ‘Attachment base only’ females laid on average 1.10 (0.29–1.91) clutches more and ‘device attached’ females laid on average 0.63 (− 0.07–1.33) clutches more than ‘non-tracked’ females (P_adj_ = 0.005 and P_adj_ = 0.084 respectively). There was, however, no significant difference in ECF between ‘device attached’ and ‘attachment base only’ females (P_adj_ = 0.492). Finally, device attachment did not have a significant effect on post-maturity growth or compound annual growth rates (Fig. 2a and b, Additional file 1: Table S3).

#### Loggerhead turtles

For loggerhead turtles, there was no significant difference in size (F_2,61_ = 1.58, *P* = 0.215, ω_p_^2^ = 0.018, Fig. 1a), mean clutch size (F_2,61_ = 0.63, *P* = 0.534, ω_p_^2^ = 0.012, Fig. 1b), RI (F_2,61_ = 0.64, *P* = 0.532, ω_p_^2^ = 0.012, Fig. 1d) and date of first nest (F_2,61_ = 1.27, *P* = 0.289, ω_p_^2^ = 0.008, Fig. 1e) between ‘attachment base only’ (*n* = 6), ‘device attached’ (*n* = 8) and ‘non-tracked’ (*n* = 50) females in the year of device attachment for female size and in the years following device attachment for reproductive correlates. Observed differences in date of first nest were due to annual effects rather than device attachment (F_1,63_ = 5.98, *P* = 0.017, ω_p_^2^ = 0.073, Fig. 1e).

However, there was a large significant difference in ECF among groups in the years following device attachment (F_2,61_ = 5.06, *P* = 0.009, ω_p_^2^ = 0.121, Fig. 1c), which could not be explained by annual effects (F_2,60_ = 0.76, *P* = 0.386, ω_p_^2^ = 0.016). ‘Attachment base only’ females laid on average 1.47 (0.09–2.85) clutches more and ‘device attached’ females laid on average 1.20 (0.03–2.37) clutches more than ‘non-tracked’ females (P_adj_ = 0.035 and P_adj_ = 0.044 respectively). There was no significant difference in ECF between the ‘device attached’ and ‘attachment base only’ females (P_adj_ = 0.921). Finally, device attachment did not have a significant effect on post-maturity growth or compound annual growth rates (Fig. 2 c and d, Additional file 1: Table S3).

### Within-female differences

**Fig. 3:**
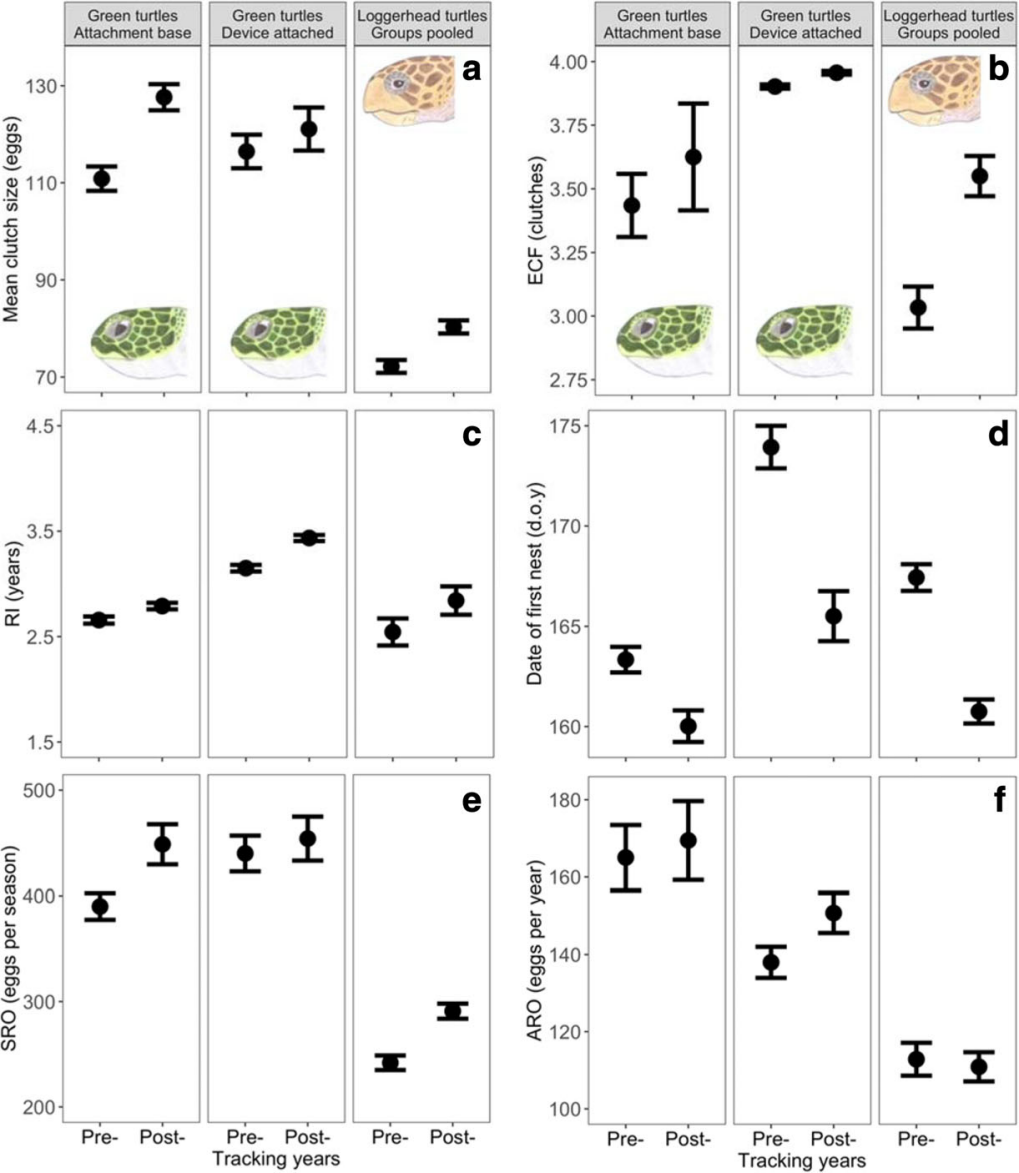
Effects on reproduction within females. Differences in mean clutch size (**a**), estimated clutch frequency (ECF, **b**), remigration interval (RI, **c**), date of first nest (d.o.y: day of the year, **d**), seasonal reproductive output (SRO, **e**) and annual reproductive output (ARO, **f**) between pre- and post-tracking years for the different groups and species. Pre-tracking years include all years including year of device attachment. Observed difference in mean clutch size (‘attachment base only’ group for green turtles and pooled group for loggerhead turtles), date of first nest (for all groups) and seasonal reproductive output (‘attachment base only’ for green turtles) between pre- and post-tracking years for particular groups were due to annual effects rather than device attachment (see Table 5). Mean ± SE

For both species, there was no significant difference in all reproductive correlates between pre- and post-tracking years (Fig. 3, Additional file 1: Table S4). Observed differences in mean clutch size and date of first nest for both species and, in seasonal reproductive output for green turtles between pre- and post-tracking years for particular groups were due to annual effects (Fig. 3, Additional file 1: Table S4). To further explore whether RI increased between pre- and post-tracking years, we looked at pairs of randomly generated consecutive RIs for ‘non-tracked’ females. We found that RI did not significantly increase between pairs for both species (green turtles: F_1,64_ = 1.64, *P* = 0.205; loggerhead turtles: F_1,30_ = 0.07, *P* = 0.798).

### Carry-over effects

**Fig. 4:**
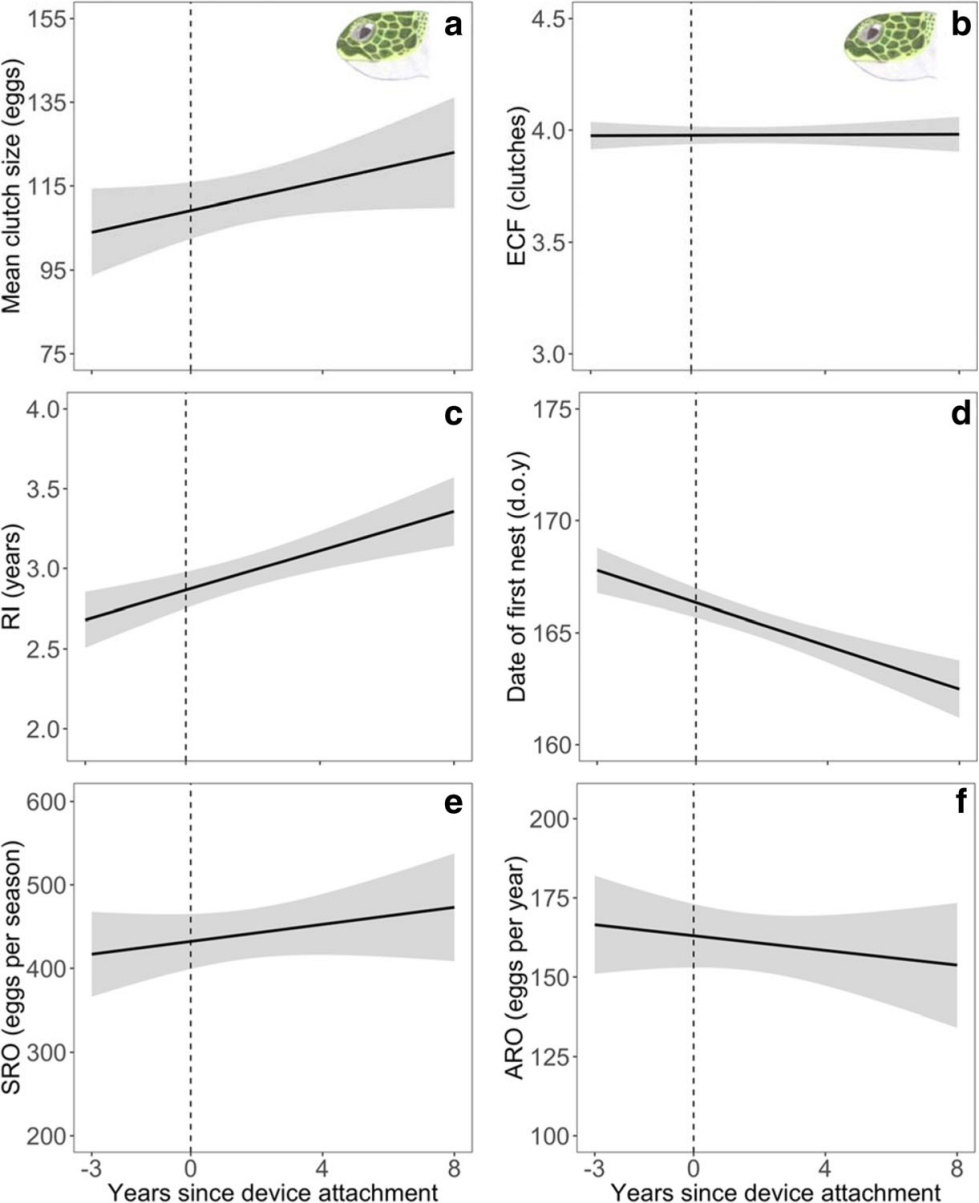
Carry-over effects on reproduction. Variation in mean clutch size (**a**), estimated clutch frequency (ECF, **b**), remigration interval (RI, **c**), date of first nest (d.o.y: day of year, **d**), seasonal reproductive output (SRO, **e**) and annual reproductive output (ARO, **f**) as a function of years since device attachment for green turtles, with year 0 being year of device attachment. The vertical dashed line represents year of device attachment. Grey shaded area represents 95% confidence intervals

For green turtles, there were no significant carry-over effects of device attachment on mean clutch size (χ^2^_1_ = 0.10, *P* = 0.756, Fig. 4a), ECF (χ^2^_1_ = 0.22, *P* = 0.639, Fig. 4b), date of first nest (χ^2^_1_ = 1.56, *P* = 0.212, Fig. 4d), seasonal reproductive output (χ^2^_1_ = 0.002, *P* = 0.963, Fig. 4e) and annual reproductive output (χ^2^_1_ = 2.84, *P* = 0.092, Fig. 4f). However, RI significantly increased by 0.67 year over the course of 4 breeding events, with device attachment occurring on the second breeding event (χ^2^_1_ = 3.93, *P* = 0.048, Fig. 4c). This increase in RI was not due to annual effects (χ^2^_1_ = 1.88, *P* = 0.171). To further explore this result, we compared RI of eight non-tracked females with similar histories across the same time frame and also found RI to increase in a similar manner (χ^2^_1_ = 6.44, *P* = 0.011).

### Survivorship

Because no estimates are available for these two populations, we had to estimate annual survival for both ‘tracked’ and ‘non-tracked’ females. Goodness of fit results and model output tables (Additional file 1: Tables S5-S8) can be found in the supplemental material for this article. All parameter estimates for both species and groups can be found in Additional file 1: Table S9.

For green turtles, annual survival was 0.91 (0.88–0.94) for ‘non-tracked’ females and 0.97 (0.95–0.99) for ‘tracked’ females. Confidence intervals were higher for ‘tracked’ females than for ‘non-tracked’ females for green turtles.

For loggerhead turtles, annual survival was 0.44 (0.30–0.61) for transient ‘non-tracked’ females, 0.83 (0.77–0.88) for remigrant ‘non-tracked’ females and 0.82 (0.73–0.90) for ‘tracked’ females. Estimates for ‘tracked’ and ‘non-tracked’ remigrant loggerhead turtles were similar, with overlapping confidence intervals.

## Discussion

Here, we provide the first analysis of the effects of device attachment on life-history traits of sea turtles. We found no evidence of deleterious effects of device attachment on reproduction, growth and annual survival of green and loggerhead turtles nesting sympatrically.

The most important effect of device attachment has been suggested to be the increase in energy expenditure, as a result of increased drag [15, 41, 53]. Sea turtles are capital breeders [54–56], meaning that the decision to nest in a given year results from the combination of an assessment of body condition and favourable environmental conditions [57]. Thus, an increase in energy expenditure during non-breeding years could have knock-on effects on the breeding phenology of study animals. Indeed, if device attachment results in reduced locomotor capacity through reduced swim speed, individuals fitted with devices at nesting grounds could arrive later at foraging grounds, but also at breeding grounds, if devices remain attached throughout the RI, which is not uncommon. In this study, as well as in previous studies [58, 59], females have been resighted at breeding grounds with devices still attached. Despite this, we found no evidence of a delayed arrival of ‘tracked’ females when subsequently returning to nest, both compared to the population at large, as well as within ‘tracked’ females. Similarly, no evidence of a delay in return rates has been observed for male loggerhead turtles [60]. In all instances, date of first nest was influenced by annual effects for both species, with first, median and final lay date having shifted towards earlier nesting over the study period [61]. Similar shifts in the breeding phenology of sea turtles have been observed in a number of populations, as a result of climate change [62–65].

In addition, device attachment could result in females requiring more time to accumulate sufficient resources to initiate reproduction [15, 20, 41, 66]. Indeed, if swimming efficiency and foraging ability are impaired by device attachment [20, 21], later arrival at foraging grounds could result in longer RIs. However, Benson et al. [67] noted that ‘tracked’ individuals arrived presumably on time at foraging grounds and we found no evidence for longer RIs in ‘tracked’ females. Although RI appeared to increase post-tracking, the magnitude of the effect was small and not significant (Fig. 3). Likewise, RI did not increase when comparing pairs of consecutive recaptures for ‘non-tracked’ females, suggesting that device attachment is unlikely to be the cause of the increase observed in ‘tracked’ females. The small sample size in this study, however, may have prevented the detection of such a trend, as a power analysis showed that a sample size of more than 400 would be needed to detect a small significant effect of device attachment on RI with a 0.8 probability. Furthermore, while RI appeared to increase with years since device attachment in green turtles, we found a similar significant increase for eight ‘non-tracked’ females with similar capture histories across the same time frame. This suggests that device attachment is unlikely to have had carry-over effects on RI of green turtles. Due to the scaling of the effects of drag on swim speed, the impacts of device attachment might be heightened in pursuit predators, such as penguins [68], and lessened in herbivores and benthic feeders, such as adult green and loggerhead turtles [69], which could explain the absence of an effect on RI. Despite the absence of a baseline for ‘non-tracked’ individuals, it is possible that changes in behaviour and swimming efficiency could have offset the effects of device attachment. Indeed, tracked females have been observed to forage ‘en route’ back to their over-wintering sites, travelling at depths and speeds which minimise drag [46, 70, 71] and thus, minimising the cost of migration and potentially device attachment.

Increased energy expenditure associated with device attachment may negatively influence reproductive output of study animals. Indeed, as females have to balance a tight energy budget, attaching devices during the inter-nesting period could have knock-on effects on their seasonal reproductive output [53]. Similarly, to overcome the increase in drag, when returning to nest, study animals either can (1) reduce their swim speed and arrive later at breeding grounds than the rest of the population, which does not appear to be the case or, (2) increase their power output thus decreasing the proportion of energy reserves available to fuel reproduction [53]. Here, we found no evidence for impaired reproductive output for either species, suggesting that females were not energetically compromised in the year of device attachment, as well as in subsequent breeding seasons. However, we cannot entirely exclude that some effects of device attachment on reproduction may have been masked by environmental effects, by the use of estimated reproductive correlates and by the targeted sampling of remigrant females, with high nest site fidelity, which can explain the differences in ECF among groups.

The cost of device attachment during the inter-nesting period will not be uniform across study animals, potentially preventing the detection of within-season effects on reproduction. While some females remain close to the nesting beach, resting on the sea floor and not actively foraging [34, 72, 73], others forage [72–74] and commute between beaches, and at times, countries, to lay their eggs [24, 39, 40]. Although it appears highly unlikely that device attachment will result in females laying fewer clutches, as improved estimates of clutch frequency have been obtained using this method, [39, 40], it will be hard to determine whether device attachment results in females laying smaller clutches due to increased energy expenditure. Depletion of resources, as shown by haematological data, is more likely to trigger the need for individuals to forage and therefore to cease reproduction [56, 75–77].

Due to the partitioning of finite resources [78], attaching devices to animals could compromise their growth, especially in juveniles, as growth is negligible in adults [44]. Nevertheless, Seney et al. [79] found no effect of device attachment on growth of captive reared juvenile individuals. Similarly, we found no evidence for such an effect in wild adult nesting green and loggerhead turtles. The significant difference in size at device attachment among groups for green turtles is likely due to targeted sampling of remigrant females in preference for some studies. Indeed, remigrant females are known to be significantly larger than neophyte nesters at Alagadi beach [44, 45] and represented the vast majority (92%) of tracked green turtles. By contrast, sampling for loggerhead turtles aimed to target females across a range of sizes [24], which resulted in a more even ratio (almost 1:1 ratio) of neophyte to remigrant nesters and can explain the absence of a significant difference in body size at device attachment among groups.

Ultimately, determining whether device attachment influences annual survival of study animals is crucial. Although device attachment has been suggested to influence return rates in some species [19, 80, 81], potentially due to increased energetic expenditure [82], we found no significant effects of device attachment on return rates or survival of ‘tracked’ females for either species.

Annual survival estimates for green turtles are not available for the Mediterranean [69], however, estimates calculated here for ‘non-tracked’ females (0.91, CI: 0.88–0.94) fall within the predictions for green turtles populations around the world (0.88, CI: 0.80–0.93). Annual survival estimates for ‘tracked’ green turtle females were higher, with non-overlapping confidence intervals, than those for ‘non-tracked’ females, which was likely due to targeted sampling of remigrant females, with higher nest site fidelity. For loggerhead turtles, our estimates for ‘non-tracked females’ (0.83, CI: 0.77–0.88) also fall within those predicted by Pfaller et al.’s [83] for loggerhead turtles around the world (0.82, CI: 0.79–0.85), are comparable to previous estimates calculated for loggerhead turtles in the Mediterranean [84, 85] and were similar to those of ‘tracked’ females in our study.

These estimates suggest that device attachment does not result in reduced annual survival in sea turtles and highlight yet again the heterogeneity of annual survival between green and loggerhead turtles worldwide, with loggerhead turtle estimates being consistently lower than those of green turtles [83]. The relatively low estimates for loggerhead turtles are thought to be linked to anthropogenic mortality, in particular bycatch, levels of which are unsustainable in the Mediterranean [86]. Finally, although annual survival estimates are prone to problems associated with tag loss [83], in this study, female identification was based on a combination of both flipper and PIT tag readings, making estimates more robust to tag loss, as PIT tag loss is thought to be negligible [87].

## Conclusion

We provide the first analysis of the effects of device attachment on life-history traits of adult sea turtles, as well as provide the first estimates of annual survival for green turtles in the Mediterranean. Although we cannot entirely exclude that small sample size, individual variation and climate change prevented the detection of an effect, device attachment was found to have no significant detrimental effects on adult sea turtles. Nevertheless, in all instances, device attachment should aim to minimise device size and drag, using low profile tags for example [88]. Finally, this study highlights the need for other similar studies elsewhere and the value of long-term individual-based monitoring.

## Additional file

Additional file 1: Additional information relating to the methods and results are provided, as well as additional Tables. **Table S1.** Significance results for return rate analysis. **Table S2.** Significance results looking at effects of device attachment on reproductive correlates among females. **Table S3.** Significance results looking at growth covariates and device attachment. **Table S4.** Significance results of within-female differences in reproductive correlates between pre- and post-tracking years. **Table S5.** Summary of models analysed in MARK for ‘non-tracked’ green turtles. **Table S6.** Summary of models analysed in MARK for ‘tracked’ green turtles. **Table S7.** Summary of models analysed in MARK for ‘non-tracked’ loggerhead turtles. **Table S8.** Summary of models analysed in MARK for ‘tracked’ loggerhead turtles. **Table S9.** Summary of parameter estimates, calculated using MARK, for both species and groups. (DOCX 50.0 kb)

## Abbreviations

ARO: Annual reproductive output
CCL: Curved carapace length
CI: Confidence interval
d.o.y: Day of the year
ECF: Estimated clutch frequency
GLMM: Generalised linear mixed model
GLS tags: Global location sensing tags
GLS: Generalised least squares
na: Not applicable
PIT tag: Passive integrated transponder tag
PTT: Platform terminal transmitters
RI: Remigration interval
SE: Standard error
SRO: Seasonal reproductive output
TDR: Time depth recorder
