# Supplementary material for: The effect of biologging systems on reproduction, growth and survival of adult sea turtles
Source: Mov Ecol. 2019 Jan 29;7:2. doi: 10.1186/s40462-018-0145-1 (PMC6350314; doi:10.1186/s40462-018-0145-1)
Supplement: Supplementary file 1 — Additional information relating to the methods and results are provided, as well as additional Tables. Table S1. Significance results for return rate analysis. Table S2. Significance results looking at effects of device attachment on reproductive correlates among females. Table S3. Significance results looking at growth covariates and device attachment. Table S4. Significance results of within-female differences in reproductive correlates between pre- and post-tracking years. Table S5. Summary of models analysed in MARK for ‘non-tracked’ green turtles. Table S6. Summary of models analysed in MARK for ‘tracked’ green turtles. Table S7. Summary of models analysed in MARK for ‘non-tracked’ loggerhead turtles. Table S8. Summary of models analysed in MARK for ‘tracked’ loggerhead turtles. Table S9. Summary of parameter estimates, calculated using MARK, for both species and groups. (DOCX 50.0 kb) [file 40462_2018_145_MOESM1_ESM.docx]

**ADDITIONAL FILE 1**

**Methods**

**Statistical analysis – Among-female differences**

Female size (curved carapace length: CCL) at device attachment or size at initial year was used to compare differences in size between ‘tracked’ and ‘non-tracked’ females. RI was calculated as the number of years elapsed between year of device attachment or initial year and the following capture. Mean clutch size, ECF and date of first nest were extracted from the following capture, i.e. post-tracking or post-initial year.

To determine whether device attachment influenced post-maturity growth, we looked at differences in curves between growth records pooled (‘tracked’ and ‘non-tracked’ females) and ‘non-tracked’ growth records, i.e. all growth records for ‘non-tracked’ females as well as growth records for ‘tracked’ females up until year of device attachment.

**Statistical analysis – Within-female differences**

To investigate within-female differences, capture histories were divided in pre- (including year of device attachment) and post-tracking years and subsequently separated in ‘attachment base only’ (n = 9) and ‘device attached’ (n = 12) groups for green turtles. Both groups were pooled (n = 10) for loggerhead turtles due to smaller sample size. If females had devices attached in more than one year, we compared the years prior and post first device attachment only. Because RI could not be calculated for pre-tracking years for females that were neophytes at device attachment, the analysis of RI and annual reproductive output only included remigrant females.

**Statistical analysis – Carry-over effects**

We included the first pre-tracking season as a baseline because devices were attached at variable points during the breeding season, which could possibly have influenced estimates of mean clutch size and ECF in the year of device attachment. We removed females that had devices attached in years following first device attachment.

**Statistical analysis – Survivorship**

Encounter histories were created for each female based on annual flipper and PIT tag re-sightings. Females were recorded as either present or absent based on successful nesting attempts. Although encounter histories are available from 1992 onwards, survey effort in 1992 was incomplete. We decided to use 1995 as the start date for the analysis of ‘non-tracked’ females as this represents three years after the beginning of the saturation tagging programme, which is equivalent to the average remigration interval for these two populations [1–3]. We therefore removed encounter histories for females that were first observed nesting prior to 1995 and after 2014 as these females will have had three or less years to potentially return to nest.

For the analysis of ‘tracked’ females, encounter histories were truncated such that the first year females were observed nesting was equivalent to the year of device attachment. This was done to avoid biasing survivorship estimates as females which had device attached as remigrants would have a survivorship equal to 1 prior to device attachment. Survivorship estimates for ‘tracked’ females will therefore reflect survival for the ‘tracked’ period rather than survival for their entire encounter history. As for ‘non-tracked’ females, we removed encounter histories for females that had devices attached after 2014. All ‘tracked’ females were included in the analysis, as females that had devices attached in multiple years will have had to have survived previous device attachments.

Survival probability was assumed to be equal in both states because separating survival probabilities between the two states would require additional data collected at foraging grounds. Encounter probability in the non-breeding state was fixed at 0. Transition probability ψ_B→NB_ was held constant for green turtles as only female was observed nesting in consecutive years at Alagadi.

Goodness of fit (GoF) was assessed using the programme U-CARE [4]. The ĉ (c-hat) estimate (quasi-likelihood over-dispersion coefficient) was calculated using U-CARE and used to adjust the model selection metric: qAIC_c_ (quasi-likelihood Akaike’s Information Criterion corrected for small sample size). Any estimates of ĉ ≤ 3.0 are considered acceptable and suggest a reasonable fit of the model to the data [5].

We used the MCMC (Markov chain Monte Carlo) method to estimate parameters because it produces unbiased estimates compared to the maximum likelihood estimation method [6]. We simulated 10 chains with 4,000 tuning samples and a burn-in period of 1,000 samples. We used 10,000 samples from the Markov chain to generate posterior distributions. We assumed prior distributions to be normal (0, 1.75) on a logit scale.

***Green turtles***

The GoF tests indicated a lack of fit of the data to the global models for ‘non-tracked’ and ‘tracked’ green turtle datasets (χ^2^_73_ = 149.95, P < 0.0001 and χ^2^_44_ = 94.46, P < 0.0001 respectively). Results from test components 3G.SR (‘non-tracked’: χ^2^_14_ = 18.28, P = 0.194; ‘tracked’: χ^2^_5_ = 4.77, P = 0.444) and M.ITEC (‘non-tracked’: χ^2^_19_ = 54.66, P < 0.0001, ‘tracked’: χ^2^_16_ = 44.57, P < 0.0001) indicated evidence of immediate trap-dependence, which can be interpreted as non-random temporary emigration [4]. A time-since-marking model structure was therefore used to estimate encounter probabilities for transient and remigrant individuals for both datasets. The over-dispersion coefficient ĉ was calculated as 2.05 for the ‘non-tracked’ dataset and as 2.15 for the ‘tracked’ dataset.

***Loggerhead turtles***

The GoF tests did not indicate a lack of fit of the data to the global models for ‘non-tracked’ and ‘tracked’ loggerhead turtle datasets (χ^2^_65_ = 70.39, P = 0.302 and χ^2^_31_ = 34.70, P = 0.296 respectively). Results from test components 3G.SR (‘non-tracked’: χ^2^_17_ = 21.67, P = 0.198; ‘tracked’: χ^2^_7_ = 2.98, P = 0.887) and M.ITEC (‘non-tracked’: χ^2^_19_ = 22.26, P = 0.272; ‘tracked’: χ^2^_12_ = 18.58, P = 0.099) did not indicate any evidence of transience or trap dependence. The over-dispersion coefficient ĉ was calculated as 1.08 for the ‘non-tracked’ dataset and as 1.12 for the ‘tracked’ dataset.

**Results**

**Survivorship**

***Green turtles***

The dataset comprised 224 encounter histories for ‘non-tracked’ green turtles and 46 encounter histories for ‘tracked’ green turtles. For both ‘non-tracked’ and ‘tracked’ datasets, the lowest qAIC_c_ ranking models estimated a single survival probability, a single recapture probability for transients and remigrant individuals and single transition probabilities. Although test components 3G.SR were not significant, we tested the most parsimonious models with a time-since-marking model structure in survival to estimate survival probabilities for transient and remigrant individuals. However, this did not result in a better fit to both datasets (see Table S5 and Table S6).

***Loggerhead turtles***

The dataset comprised 327 encounter histories for ‘non-tracked’ loggerhead turtles and 46 encounter histories for ‘tracked’ loggerhead turtles. Due to the small size of the ‘non-tracked’ loggerhead turtle dataset and the small number of females that returned to nest following device attachment, transition probabilities were held constant over time for the ‘non-tracked’ dataset. For both ‘tracked’ and ‘non-tracked’ datasets, the lowest qAIC_c_ ranking models estimated a single survival probability, a single recapture probability and single transition probabilities. Although test components 3G.SR and M.ITEC were not significant, we tested the most parsimonious models with a time-since-marking model structure in survival and recapture probability to estimate probabilities for transient and remigrant individuals, as a large number of females nesting at Alagadi can be considered transients. The model accounting for transience only resulted in a better fit to the data than the other models for the ‘non-tracked’ dataset (see Table S7). However, it did not result in a better fit to the data for the ‘tracked’ dataset (see Table S8).

**References**

1. Broderick AC, Glen F, Godley BJ, Hays GC. Variation in reproductive output of marine turtles. J Exp Mar Biol Ecol. 2003;288:95–109.

2. Stokes KL, Fuller WJ, Glen F, Godley BJ, Hodgson DJ, Rhodes KA, et al. Detecting green shoots of recovery: The importance of long-term individual-based monitoring of marine turtles. Anim Conserv. 2014;17:593–602.

3. Omeyer LCM, Fuller WJ, Godley BJ, Snape RTE, Broderick AC. Determinate or indeterminate growth ? Revisiting the growth strategy of sea turtles. Mar Ecol Prog Ser. 2018;596:199–211.

4. Choquet R, Reboulet A-M, Lebreton JD, Gimenez O, Pradel R. U- CARE 2.2 User’s Manual. Montpellier (France); 2005.

5. Lebreton JD, Burnham KP, Clobert J, Anderson DR. Modeling survival and testing biological hypotheses using marked animals: a unified approach with case studies. Ecol Monogr. 1992;62:67–118.

6. Link W, Cam E, Nichols J, Cooch E. Of BUGS and birds: Markov chain Monte Carlo for hierarchical modeling in wildlife research. J Wildl Manag. 2002;66:277–91.

**Table S1. Significance results for return rate analysis**.

| **Species** | **Timeframe** | **Test 1: tracked versus**  **non-tracked neophytes** | | **Test 2: tracked neophytes versus tracked remigrants** | | **Test 3: ‘attachment base only versus ‘device attached’ groups** | |
| --- | --- | --- | --- | --- | --- | --- | --- |
|  |  | p value | Odds ratio (95 % CI) | p value | Odds ratio (95 % CI) | p value | Odds ratio (95 % CI) |
| Green turtles | ≤ 5 yr | 0.271 | 0.383 (0.090 – 1.643) | 0.382 | 0.460 (0.090 – 2.350) | 0.310 | 0.403 (0.060 – 1.991) |
|  | ≤ 10 yr | 0.282 | 0.344 (0.068 – 1.744) | 0.574 | 0.387 (0.057 – 2.612) | 0.678 | 0.595 (0.048 – 4.763) |
|  | ≤ 15 yr | 0.127 | 0.187 (0.022 – 1.586) | 1.000 | 0.387 (0.030 – 4.981) | 0.596 | 2.304 (0.111 – 145.491) |
| Loggerhead turtles | ≤ 5 yr | 0.407 | 0.529 (0.130 – 2.163) | 0.440 | 0.467 (0.059 – 2.930) | 0.440 | 2.143 (0.341 – 16.895) |
|  | ≤ 10 yr | 0.696 | 0.678 (0.159 – 2.895) | 0.678 | 0.504 (0.061 – 3.452) | 0.678 | 1.986 (0.290 – 16.454) |
|  | ≤ 15 yr | 0.343 | 0.343 (0.056 – 2.086) | 0.613 | 0.563 (0.044 – 9.110) | 0.613 | 1.775 (0.110 – 22.859) |

Test 1 investigates whether there is a significant difference between the number of resighted ‘tracked’ and ‘non-tracked’ neophyte (first-time nesters) females. Test 2 investigates whether there is a significant difference between the number of resighted ‘tracked’ neophyte and remigrant females. Finally, test 3 investigates whether there is a significant difference between the number of resighted ‘attachment base only’ and ‘device attached’ females. Odds ratio are used as a measure of effect size. CI: confidence intervals.

**Table S2. Significance results looking at effects of device attachment on reproductive correlates among females.**

| **Species** | | **CCL**  **(cm)** | **Mean clutch size**  **(eggs)** | **ECF**  **(clutches)** | **RI**  **(years)** | **Date of first nest**  **(day of year)** |
| --- | --- | --- | --- | --- | --- | --- |
|  | **Group** |  |  |  |  |  |
| Green turtles | |  |  |  |  |  |
|  | ‘Non-tracked’  (n = 94) | 87.1 ± 5.7  (74.3 – 99.0) | 115.9 ± 20.3  (69.0 – 184.0) | 3.0 ± 1.3 (1.0 – 6.0) | 3.7 ± 1.5  (2.0 – 9.0) | 167.1 ± 14.6  (142.0 – 224.0) |
|  | ‘Attachment base only’  (n = 14) | 90.1 ± 6.2  (81.5 – 103.0) | 116.8 ± 25.6  (72.0 – 162.0) | 4.1 ± 1.0 (2.0 – 6.0) | 3.4 ± 1.6  (2.0 – 8.0) | 159.6 ± 7.1  (151.0 – 175.0) |
|  | ‘Device attached’  (n = 20) | 93.1 ± 6.4  (79.7 – 105.0) | 117.0 ± 26.6  (62.0 – 154.0) | 3.6 ± 0.9 (1.0 – 5.0) | 3.9 ± 2.0  (2.0 – 10.0) | 167.2 ± 10.8  (144.0 – 186.0) |
| Loggerhead turtles | |  |  |  |  |  |
|  | ‘Non-tracked’  (n = 50) | 73.2 ± 3.7  (65.4 – 82.0) | 78.8 ± 15.9  (47.5 – 124.0) | 2.3 ± 1.3 (1.0 – 5.0) | 3.8 ± 2.0  (1.0 – 10.0) | 165.7 ± 13.0  (147.0 – 198.0) |
|  | ‘Attachment base only’  (n = 6) | 76.4 ± 3.1  (72.5 – 81.2) | 85.5 ± 10.3  (76.0 – 99.0) | 3.7 ± 0.8 (3.0 – 5.0) | 3.5 ± 0.8  (3.0 – 5.0) | 154.2 ± 9.2  (143.0 – 170.0) |
|  | ‘Device attached’  (n = 8) | 74.1 ± 6.9  (67.5 – 87.2) | 74.9 ± 10.7  (64.0 – 99.0) | 3.5 ± 1.7 (1.0 – 6.0) | 2.9 ± 1.0  (2.0 – 5.0) | 161.5 ± 12.8  (142.0 – 187.0) |

Differences in body size in the year of device attachment and reproductive correlates in the years following device attachment among groups of females. For ‘non-tracked’ females, the year(s) of and following device attachment represent randomly generated following recaptures. Mean ± SD (range). CCL: curved carapace length; ECF: estimated clutch frequency; RI: remigration interval.

**Table S3. Significance results looking at growth covariates and device attachment.**

| **Species** | **Model** | **Interaction** | **Coefficient ( ± SE)** | **t value** | **p value** |
| --- | --- | --- | --- | --- | --- |
| Green turtles | Annual growth | Years since first capture : Tracking | 0.002 ± 0.014 | 0.167 | 0.867 |
|  |  | Mean CCL : Tracking | -0.002 ± 0.002 | -1.086 | 0.278 |
|  |  | RI : Tracking | 0.029 ± 0.043 | 0.660 | 0.510 |
|  | Compound annual growth rates | Years since first capture : Tracking | -0.013 ± 0.014 | -0.915 | 0.361 |
|  |  | RI : Tracking | 0.025 ± 0.042 | 0.590 | 0.556 |
| Loggerhead turtles | Annual growth | Years since first capture : Tracking | 0.013 ± 0.031 | 0.422 | 0.674 |
|  |  | Mean CCL : Tracking | 0.001 ± 0.007 | 0.204 | 0.839 |
|  |  | RI : Tracking | -0.004 ± 0.142 | -0.026 | 0.980 |
|  | Compound annual growth rates | Years since first capture : Tracking | 0.025 ± 0.038 | 0.673 | 0.502 |
|  |  | RI : Tracking | 0.012 ± 0.127 | 0.097 | 0.923 |

Growth covariates are those used in Omeyer et al. [3] to investigate whether device attachment influenced post-maturity growth of ‘tracked’ females. SE: standard error; CCL: curved carapace length; CAGR: compound annual growth rates.

**Table S4.** **Significance results of** **within-female differences in reproductive correlates between pre- and post-tracking years.**

| **Species** | **Group** | **Significance: reproduction** | | | **Significance: annual effect** | |
| --- | --- | --- | --- | --- | --- | --- |
|  |  | **Reproductive correlate** | **χ^2^_1_** | **p value** | **χ^2^_1_** | **p value** |
| Green turtles | ‘Attachment base only’ | *Mean clutch size* | 0.13 | 0.716 | *11.24* | *< 0.0001* |
|  |  | Estimated clutch frequency | 0.87 | 0.351 | 0.03 | 0.861 |
|  |  | Remigration interval | 0.31 | 0.579 | 0.66 | 0.415 |
|  |  | *Date of first nest* | 0.19 | 0.662 | *5.39* | *0.020* |
|  |  | *Seasonal reproductive output* | 0.99 | 0.320 | *5.10* | *0.024* |
|  |  | Annual reproductive output | 1.41 | 0.235 | 0.04 | 0.844 |
|  | ‘Device attached’ | Mean clutch size | 0.01 | 0.927 | 1.63 | 0.202 |
|  |  | Estimated clutch frequency | 0.07 | 0.789 | 0.05 | 0.821 |
|  |  | Remigration interval | 0.03 | 0.867 | 1.77 | 0.184 |
|  |  | *Date of first nest* | < 0.001 | 0.987 | *10.05* | *0.002* |
|  |  | Seasonal reproductive output | 0.10 | 0.755 | 0.83 | 0.361 |
|  |  | Annual reproductive output | 0.64 | 0.425 | 0.01 | 0.909 |
| Loggerhead turtles | Groups pooled | *Mean clutch size* | 0.24 | 0.625 | *4.72* | *0.030* |
|  |  | Estimated clutch frequency | 3.44 | 0.064 | 0.21 | 0.643 |
|  |  | Remigration interval | 2.04 | 0.153 | 1.05 | 0.305 |
|  |  | *Date of first nest* | 0.08 | 0.780 | *7.69* | *0.006* |
|  |  | Seasonal reproductive output | 2.63 | 0.105 | 1.27 | 0.260 |
|  |  | Annual reproductive output | 0.55 | 0.460 | 0.43 | 0.513 |

‘Attachamen base only’ and ‘device attached’ groups were pooled for loggerhead turtles due to smaller sample size. Significant results are italicised.

**Table S5**. **Summary of models analysed in MARK for ‘non-tracked’ green turtles.**

| **Model** | **qAICc** | **ΔqAICc** | **AICc weights** | **Model likelihood** |
| --- | --- | --- | --- | --- |
| S(.) p_B_(tsm–./.) p_NB_(0) ψ_B→NB_(.) ψ_NB→B_(.) | 445.57 | 0.00 | 0.999 | 0.999 |
| S(tsm–./.) p_B_(tsm–./.) p_NB_(0) ψ_B→NB_(.) ψ_NB→B_(.) | 459.43 | 13.87 | 0.001 | 0.001 |
| S(.) p_B_(tsm–./t) p_NB_(0) ψ_B→NB_(.) ψ_NB→B_(.) | 474.21 | 28.64 | 0.000 | 0.000 |
| S(.) p_B_(tsm–./.) p_NB_(0) ψ_B→NB_(.) ψ_NB→B_(t) | 474.71 | 29.14 | 0.000 | 0.000 |
| S(t) p_B_(tsm–./.) p_NB_(0) ψ_B→NB_(.) ψ_NB→B_(.) | 481.29 | 35.72 | 0.000 | 0.000 |
| S(.) p_B_(tsm–t/.) p_NB_(0) ψ_B→NB_(.) ψ_NB→B_(.) | 484.88 | 39.32 | 0.000 | 0.000 |
| S(.) p_B_(tsm–./t) p_NB_(0) ψ_B→NB_(.) ψ_NB→B_(t) | 504.89 | 59.32 | 0.000 | 0.000 |
| S(t) p_B_(tsm–./t) p_NB_(0) ψ_B→NB_(.) ψ_NB→B_(.) | 509.18 | 63.61 | 0.000 | 0.000 |
| S(t) p_B_(tsm–./.) p_NB_(0) ψ_B→NB_(.) ψ_NB→B_(t) | 517.70 | 72.14 | 0.000 | 0.000 |
| S(.) p_B_(tsm–t/t) p_NB_(0) ψ_B→NB_(.) ψ_NB→B_(.) | 518.29 | 72.72 | 0.000 | 0.000 |
| S(.) p_B_(tsm–t/.) p_NB_(0) ψ_B→NB_(.) ψ_NB→B_(t) | 519.67 | 74.10 | 0.000 | 0.000 |
| S(t) p_B_(tsm–t/.) p_NB_(0) ψ_B→NB_(.) ψ_NB→B_(.) | 526.50 | 80.94 | 0.000 | 0.000 |
| S(t) p_B_(tsm–./t) p_NB_(0) ψ_B→NB_(.) ψ_NB→B_(t) | 552.70 | 107.13 | 0.000 | 0.000 |
| S(.) p_B_(tsm–t/t) p_NB_(0) ψ_B→NB_(.) ψ_NB→B_(t) | 555.77 | 110.21 | 0.000 | 0.000 |
| S(t) p_B_(tsm–t/.) p_NB_(0) ψ_B→NB_(.) ψ_NB→B_(t) | 569.71 | 124.15 | 0.000 | 0.000 |
| S(t) p_B_(tsm–t/t) p_NB_(0) ψ_B→NB_(.) ψ_NB→B_(.) | 574.13 | 128.56 | 0.000 | 0.000 |
| S(t) p_B_(tsm–t/t) p_NB_(0) ψ_B→NB_(.) ψ_NB→B_(t) | 612.08 | 166.52 | 0.000 | 0.000 |

qAIC_c_: quasi-likelihood Akaike’s information criterion corrected for small sample size; S: survival rate, assumed to be equal in the breeding and non-breeding state; p_B_: probability of recapture in the breeding state; p_NB_: probability of recapture in the non-breeding state, fixed at 0 throughout; ψ_B→NB_: breeding transition probability from the breeding to the non-breeding state, held constant as green turtles extremely rarely breed in consecutive years; ψ_NB→B_: breeding transition probability from the non-breeding to the breeding state; ‘t’ denotes that the parameter was time varying; ‘.’ denotes that the parameter was constant; ‘tsm’: time-since-marking model structure; ‘/’ separates age-classes (transients versus remigrants) for parameters with a time-since-marking model structure.

**Table S6**. **Summary of models analysed in MARK for ‘tracked’ green turtles.**

| **Model** | **qAIC_c_** | **ΔqAICc** | **AIC_c_ weights** | **Model likelihood** |
| --- | --- | --- | --- | --- |
| S(.) p_B_(tsm–./.) p_NB_(0) ψ_B→NB_(.) ψ_NB→B_(.) | 209.33 | 0.00 | 0.695 | 1.000 |
| S(tsm–./.) p_B_(tsm–./.) p_NB_(0) ψ_B→NB_(.) ψ_NB→B_(.) | 210.99 | 1.67 | 0.302 | 0.434 |
| S(.) p_B_(tsm–./t) p_NB_(0) ψ_B→NB_(.) ψ_NB→B_(.) | 221.92 | 12.60 | 0.001 | 0.002 |
| S(.) p_B_(tsm–t/t) p_NB_(0) ψ_B→NB_(.) ψ_NB→B_(.) | 221.92 | 12.60 | 0.001 | 0.002 |
| S(.) p_B_(tsm–t/.) p_NB_(0) ψ_B→NB_(.) ψ_NB→B_(.) | 225.42 | 16.09 | 0.000 | 0.000 |
| S(.) p_B_(tsm–./.) p_NB_(0) ψ_B→NB_(.) ψ_NB→B_(t) | 239.67 | 30.34 | 0.000 | 0.000 |
| S(.) p_B_(tsm–t/.) p_NB_(0) ψ_B→NB_(.) ψ_NB→B_(t) | 239.67 | 30.34 | 0.000 | 0.000 |
| S(t) p_B_(tsm–./.) p_NB_(0) ψ_B→NB_(.) ψ_NB→B_(.) | 251.96 | 42.64 | 0.000 | 0.000 |
| S(t) p_B_(tsm–t/.) p_NB_(0) ψ_B→NB_(.) ψ_NB→B_(.) | 251.96 | 42.64 | 0.000 | 0000 |
| S(.) p_B_(tsm–./t) p_NB_(0) ψ_B→NB_(.) ψ_NB→B_(t) | 257.21 | 47.88 | 0.000 | 0.000 |
| S(.) p_B_(tsm–t/t) p_NB_(0) ψ_B→NB_(.) ψ_NB→B_(t) | 257.21 | 47.88 | 0.000 | 0.000 |
| S(t) p_B_(tsm–./t) p_NB_(0) ψ_B→NB_(.) ψ_NB→B_(.) | 272.73 | 63.41 | 0.000 | 0.000 |
| S(t) p_B_(tsm–./.) p_NB_(0) ψ_B→NB_(.) ψ_NB→B_(t) | 293.80 | 84.48 | 0.000 | 0.000 |
| S(t) p_B_(tsm–t/.) p_NB_(0) ψ_B→NB_(.) ψ_NB→B_(t) | 293.80 | 84.48 | 0.000 | 0.000 |
| S(t) p_B_(tsm–./t) p_NB_(0) ψ_B→NB_(.) ψ_NB→B_(t) | 321.36 | 112.04 | 0.000 | 0.000 |
| S(t) p_B_(tsm–t/t) p_NB_(0) ψ_B→NB_(.) ψ_NB→B_(t) | 321.36 | 112.04 | 0.000 | 0.000 |
| S(t) p_B_(tsm–t/t) p_NB_(0) ψ_B→NB_(.) ψ_NB→B_(.) | 327.22 | 117.89 | 0.000 | 0.000 |

qAIC_c_: quasi-likelihood Akaike’s information criterion corrected for small sample size; S: survival rate, assumed to be equal in the breeding and non-breeding state; p_B_: probability of recapture in the breeding state; p_NB_: probability of recapture in the non-breeding state, fixed at 0 throughout; ψ_B→NB_: breeding transition probability from the breeding to the non-breeding state, held constant as green turtles extremely rarely breed in consecutive years; ψ_NB→B_: breeding transition probability from the non-breeding to the breeding state; ‘t’ denotes that the parameter was time varying; ‘.’ denotes that the parameter was constant; ‘tsm’: time-since-marking model structure; ‘/’ separates age-classes (individuals that become transient after device attachment and individuals that remain in the breeding population after device attachment) for parameters with a time-since-marking model structure.

**Table S7**. **Summary of models analysed in MARK for ‘non-tracked’ loggerhead turtles.**

| **Model** | **qAICc** | **ΔqAICc** | **AICc weights** | **Model likelihood** |
| --- | --- | --- | --- | --- |
| S(tsm–./.) p_B_(.) p_NB_(0) ψ_B→NB_(.) ψ_NB→B_(.) | 667.06 | 0.00 | 0.705 | 1.000 |
| S(tsm–./.) p_B_(tsm–./.) p_NB_(0) ψ_B→B_(.) ψ_NB→B_(.) | 669.11 | 2.05 | 0.253 | 0.359 |
| S(.) p_B_(.) p_NB_(0) ψ_B→NB_(.) ψ_NB→B_(.) | 673.79 | 6.72 | 0.024 | 0.035 |
| S(.) p_B_(tsm–./.) p_NB_(0) ψ_B→NB_(.) ψ_NB→B_(.) | 674.96 | 7.90 | 0.014 | 0.019 |
| S(.) p_B_(t) p_NB_(0) ψ_B→NB_(.) ψ_NB→B_(.) | 678.41 | 11.35 | 0.002 | 0.003 |
| S(.) p_B_(.) p_NB_(0) ψ_B→NB_(.) ψ_NB→B_(t) | 679.25 | 12.19 | 0.002 | 0.002 |
| S(.) p_B_(.) p_NB_(0) ψ_B→NB_(t) ψ_NB→B_(.) | 691.29 | 24.23 | 0.000 | 0.000 |
| S(t) p_B_(.) p_NB_(0) ψ_B→NB_(.) ψ_NB→B_(.) | 698.59 | 31.53 | 0.000 | 0.000 |
| S(.) p_B_(t) p_NB_(0) ψ_B→NB_(t) ψ_NB→B_(.) | 705.68 | 38.62 | 0.000 | 0.000 |
| S(.) p_B_(t) p_NB_(0) ψ_B→NB_(.) ψ_NB→B_(t) | 705.73 | 38.66 | 0.000 | 0.000 |
| S(.) p_B_(.) p_NB_(0) ψ_B→NB_(t) ψ_NB→B_(t) | 712.35 | 45.29 | 0.000 | 0.000 |
| S(t) p_B_(t) p_NB_(0) ψ_B→NB_(.) ψ_NB→B_(.) | 714.38 | 47.32 | 0.000 | 0.000 |
| S(t) p_B_(.) p_NB_(0) ψ_B→NB_(.) ψ_NB→B_(t) | 716.21 | 49.14 | 0.000 | 0.000 |
| S(t) p_B_(.) p_NB_(0) ψ_B→NB_(t) ψ_NB→B_(.) | 728.82 | 61.76 | 0.000 | 0.000 |
| S(.) p_B_(t) p_NB_(0) ψ_B→NB_(t) ψ_NB→B_(t) | 736.28 | 69.21 | 0.000 | 0.000 |
| S(t) p_B_(t) p_NB_(0) ψ_B→NB_(t) ψ_NB→B_(.) | 744.28 | 77.21 | 0.000 | 0.000 |
| S(t) p_B_(t) p_NB_(0) ψ_B→NB_(.) ψ_NB→B_(t) | 745.50 | 78.44 | 0.000 | 0.000 |
| S(t) p_B_(.) p_NB_(0) ψ_B→NB_(t) ψ_NB→B_(t) | 749.32 | 82.26 | 0.000 | 0.000 |
| S(t) p_B_(t) p_NB_(0) ψ_B→NB_(t) ψ_NB→B_(t) | 773.14 | 106.08 | 0.000 | 0.000 |

qAIC_c_: quasi-likelihood Akaike’s information criterion corrected for small sample size; S: survival rate, assumed to be equal in the breeding and non-breeding state; p_B_: probability of recapture in the breeding state; p_NB_: probability of recapture in the non-breeding state, fixed at 0 throughout; ψ_B→NB_: breeding transition probability from the breeding to the non-breeding state, held constant as green turtles extremely rarely breed in consecutive years; ψ_NB→B_: breeding transition probability from the non-breeding to the breeding state; ‘t’ denotes that the parameter was time varying; ‘.’ denotes that the parameter was constant; ‘tsm’: time-since-marking model structure; ‘/’ separates age-classes (transients versus remigrants) for parameters with a time-since-marking model structure.

**Table S8**. **Summary of models analysed in MARK for ‘tracked’ loggerhead turtles.**

| **Model** | **qAICc** | **ΔqAICc** | **AICc weights** | **Model likelihood** |
| --- | --- | --- | --- | --- |
| S(.) p_B_(.) p_NB_(0) ψ_B→NB_(.) ψ_NB→B_(.) | 152.56 | 0.00 | 0.414 | 1.000 |
| S(tsm–./.) p_B_(.) p_NB_(0) ψ_B→NB_(.) ψ_NB→B_(.) | 152.89 | 0.32 | 0.352 | 0.850 |
| S(.) p_B_(tsm–./.) p_NB_(0) ψ_B→NB_(.) ψ_NB→B_(.) | 154.89 | 2.33 | 0.129 | 0.312 |
| S(tsm–./.) p_B_(tsm–./.) p_NB_(0) ψ_B→NB_(.) ψ_NB→B_(.) | 155.29 | 2.73 | 0.106 | 0.256 |
| S(.) p_B_(t) p_NB_(0) ψ_B→NB_(.) ψ_NB→B_(.) | 188.90 | 36.34 | 0.000 | 0.000 |
| S(t) p_B_(.) p_NB_(0) ψ_B→NB_(.) ψ_NB→B_(.) | 194.75 | 42.19 | 0.000 | 0.000 |
| S(t) p_B_(t) p_NB_(0) ψ_B→NB_(.) ψ_NB→B_(.) | 275.30 | 122.74 | 0.000 | 0.000 |

Due to the small size of the dataset and the small number of females that returned to nest following device attachment, transition probabilities were held constant over time. qAIC_c_: quasi-likelihood Akaike’s information criterion corrected for small sample size; S: survival rate, assumed to be equal in the breeding and non-breeding state; p_B_: probability of recapture in the breeding state; p_NB_: probability of recapture in the non-breeding state, fixed at 0 throughout; ψ_B→NB_: breeding transition probability from the breeding to the non-breeding state; ψ_NB→B_: breeding transition probability from the non-breeding to the breeding state; ‘.’ denotes that the parameter was constant; ‘tsm’: time-since-marking model structure; ‘/’ separates age-classes (individuals that become transient after device attachment and individuals that remain in the breeding population after device attachment) for parameters with a time-since-marking model structure.

**Table S9**. **Summary of parameter estimates, calculated using MARK, for both species and groups.**

| **Species** | | **Survival** | **Recapture probability**  **in the breeding state** | **Breeding transition probability between states** |
| --- | --- | --- | --- | --- |
|  | **Group** |  |  |  |
| Green turtles | |  |  |  |
|  | ‘Non-tracked’ | S = 0.91 (0.88 – 0.94) | p_B_ (transients) = 0.39 (0.02 – 0.89)  p_B_ (remigrants) = 0.87 (0.70 – 1.00) | ψ_B→NB_ = 0.98 (0.95 – 1.00)  ψ_NB→B_ = 0.22 (0.16 – 0.30) |
|  | ‘Tracked’ | S = 0.97 (0.95 – 0.99) | p_B_ (transients) = 0.43 (0.01 – 0.93)  p_B_ (remigrants) = 0.92 (0.79 – 1.00) | ψ_B→NB_ = 0.98 (0.96 – 1.00)  ψ_NB→B_ = 0.35 (0.27 – 0.43) |
| Loggerhead turtles | |  |  |  |
|  | ‘Non-tracked’ | S (transients) = 0.44 (0.30 – 0.61)  S (remigrants) = 0.83 (0.77 – 0.88) | p_B_ = 0.55 (0.23 – 0.99) | ψ_B→NB_ = 0.89 (0.74 – 0.98)  ψ_NB→B_ = 0.37 (0.11 – 0.73) |
|  | ‘Tracked’ | S = 0.82 (0.73 – 0.90) | p_B_ = 0.78 (0.48 – 1.00) | ψ_B→NB_ = 0.94 (0.87 – 1.00)  ψ_NB→B_ = 0.38 (0.20 – 0.60) |

Survival, recapture probability and breeding transition probability: mean (95 % highest posterior density confidence intervals). ψ_B→NB_: breeding transition probability from the breeding to the non-breeding state; ψ_NB→B_: breeding transition probability from the non-breeding to the breeding state.
